# Wearability Testing of Ambulatory Vital Sign Monitoring Devices: Prospective Observational Cohort Study

**DOI:** 10.2196/20214

**Published:** 2020-12-16

**Authors:** Carlos Areia, Louise Young, Sarah Vollam, Jody Ede, Mauro Santos, Lionel Tarassenko, Peter Watkinson

**Affiliations:** 1 Critical Care Research Group Nuffield Department of Clinical Neurosciences University of Oxford Oxford United Kingdom; 2 National Institute for Health Research Biomedical Research Centre Oxford United Kingdom; 3 Department of Engineering Science Institute of Biomedical Engineering University of Oxford Oxford United Kingdom

**Keywords:** wearables, pulse oximeter, chest patch, wearability, vital signs, ambulatory monitoring

## Abstract

**Background:**

Timely recognition of patient deterioration remains challenging. Ambulatory monitoring systems (AMSs) may provide support to current monitoring practices; however, they need to be thoroughly tested before implementation in the clinical environment for early detection of deterioration.

**Objective:**

The objective of this study was to assess the wearability of a selection of commercially available AMSs to inform a future prospective study of ambulatory vital sign monitors in an acute hospital ward.

**Methods:**

Five pulse oximeters (4 with finger probes and 1 wrist-worn only, collecting pulse rates and oxygen saturation) and 2 chest patches (collecting heart rates and respiratory rates) were selected to be part of this study: The 2 chest-worn patches were VitalPatch (VitalConnect) and Peerbridge Cor (Peerbridge); the 4 wrist-worn devices with finger probe were Nonin WristOx2 3150 (Nonin), Checkme O2+ (Viatom Technology), PC-68B, and AP-20 (both from Creative Medical); and the 1 solely wrist-worn device was Wavelet (Wavelet Health). Adult participants wore each device for up to 72 hours while performing usual “activities of daily living” and were asked to score the perceived exertion and perception of pain or discomfort by using the Borg CR-10 scale; thoughts and feelings caused by the AMS using the Comfort Rating Scale (CRS); and to provide general free text feedback. Median and IQRs were reported and nonparametric tests were used to assess differences between the devices’ CRS scores.

**Results:**

Quantitative scores and feedback were collected in 70 completed questionnaires from 20 healthy volunteers, with each device tested approximately 10 times. The Wavelet seemed to be the most wearable device (*P*<.001) with an overall median (IQR) CRS score of 1.00 (0.88). There were no statistically significant differences in wearability between the chest patches in the CRS total score; however, the VitalPatch was superior in the Attachment section (*P*=.04) with a median (IQR) score of 3.00 (1.00). General pain and discomfort scores and total percentage of time worn are also reflective of this.

**Conclusions:**

Our results suggest that adult participants prefer to wear wrist-worn pulse oximeters without a probe compressing the fingertip and they prefer to wear a smaller chest patch. A compromise between wearability, reliability, and accuracy should be made for successful and practical integration of AMSs within the hospital environment.

## Introduction

### Background

Failure to recognize and act on deteriorating signs of acute illness has been documented previously [1,2]. The National Institute for Health and Care Excellence [3] recommends the use of an early warning score, which is designed to quantitatively assess the severity of abnormal vital signs triggering the appropriately graded clinical response. A limitation of early warning score systems is the requirement for clinical staff to measure vital signs at the correct frequency. There are several factors that can affect monitoring frequency such as clinical shift duration [4], ward staff levels [5] and workload associated with the vital sign measurements [6]; hence, the ideal frequency is often not achieved [7-9].

Research has shown that the current ward-based vital-sign monitoring of patients is time consuming, as there can be several processes involved in addition to manual measurement, for example, explaining the process and obtaining consent from patients, documenting vital signs in patient records, calculating the early warning score, among others [6,10]. Additionally, even if the ideal frequency of measurement is achieved, patients might deteriorate between observation sets [11].

To address this, patients could be continuously monitored with the aim of increasing early detection of deterioration [12]. In the United Kingdom, continuous monitoring is undertaken in clinical practice but is not commonly performed in wards [13]. It has also been suggested that the most frequent reason for nonuse of continuous monitoring systems is restriction of patient movement and that, to maximize clinical integration, continuous monitoring should be comfortable and less restrictive [13,14].

Ambulatory monitoring systems (AMSs) may provide an alternative to either intermittent measurement of manual vital signs or wired continuous monitoring, affording the patients more mobility and comfort while supporting clinical staff by providing regular vital signs data [15]. There is an increased focus on the development of wireless vital sign monitors for use in the health care setting; however, their reliability and efficiency are still uncertain and need to be tested [16]. Additionally, it has been suggested that introducing AMSs may have physical or psychological effects that should be assessed [17-19] in order to maximize patient compliance and data retrieval. Previous studies have shown that AMSs are removed prematurely owing to patient irritation, discomfort, feeling unwell, or equipment failure [20].

This is the first study of our virtual high dependency unit project, with the overall aim of testing the feasibility of deploying ambulatory vital sign monitoring in the hospital environment. To achieve this, and considering the nonadoption, abandonment, scale-up, spread, and sustainability framework [21], this study will support the initial AMS selection to move into further testing within the virtual high dependency unit project. This will include ward locational testing as well as tests of device accuracy during patient movement and in the detection of hypoxia [22]. Once the final devices have been selected, we will integrate these within our user interface and test its clinical deployment.

### Objective

The aim of this study was to assess the wearability of a selection of commercially available AMSs to inform a future prospective study of wearable vital sign monitors in an acute hospital ward.

## Methods

### Device Selection

**Figure 1:**
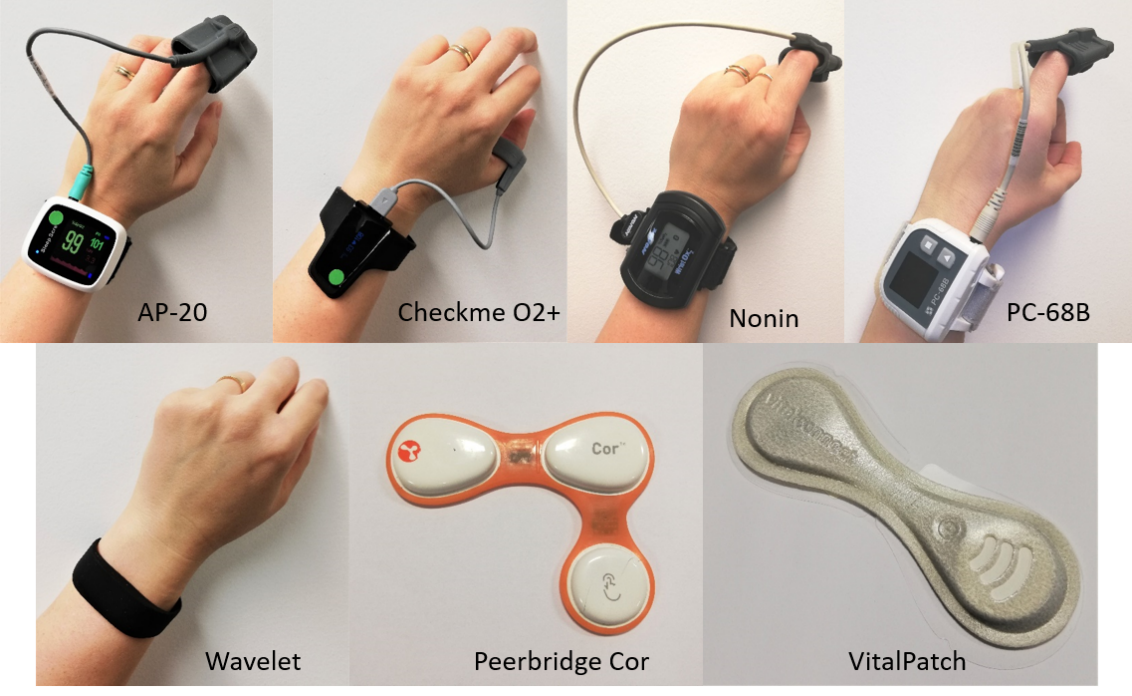
Devices included in this study.

The research team conducted a preliminary review of commercially available ambulatory vital sign monitors. To be considered for this study, the devices were required to have wireless connectivity, to measure at least two of the target parameters (ie, heart rate, oxygen saturation, respiratory rate) and to provide third-party permission to access raw data. Based on these requirements, we selected the following monitors: 2 chest-worn patches, that is, VitalPatch (VitalConnect) and Peerbridge Cor (Peerbridge); 4 wrist-worn devices with finger probe, that is, Nonin WristOx2 3150 (Nonin), Checkme O2+ (Viatom Technology), PC-68B, and AP-20 (both from Creative Medical); and 1 solely wrist-worn device, that is, Wavelet (Wavelet Health). Nonin WristOx2 3150 is named Nonin hereafter (Figure 1).

### Study Design and Participants

This study used a prospective observational cohort design. It was reviewed and approved by the Oxford University Research and Ethics Committee and Clinical Trials and Research Governance teams (R55430/RE003). This study is compliant with the cohort checklist of the STROBE (STrengthening the Reporting of OBservational studies in Epidemiology) statement Multimedia Appendix 1.

Adult participants were in-house research staff based in the Kadoorie Research Centre (Level 3, John Radcliffe Hospital) and the Oxford Institute of Biomedical Engineering (University of Oxford, Old Road Campus Research Building), who were recruited through posters placed in target locations such as office common spaces. We also used the internal departmental newsletter and distributed the participant information sheet within the departments. Once a volunteer expressed interest in the study via email/telephone, a study session was organized. All participants were healthy adult volunteers, with the only exclusion criterion being known allergies to adhesive stickers or intracardiac devices (permanent pacemaker).

Following informed consent, participants had at least one of the AMSs fitted and were guided through the device practicalities (eg, how to remove and reattach, waterproofing advice). Participants were required to wear up to 4 different AMSs for up to 72 hours each to mimic in-hospital use. They were advised that they could remove the device if desired; they were then requested to log the time and reason for removal (eg, not being able to wear the finger probe while cooking). Participants were also asked to score various activities that patients are likely to perform during their hospital stay by using a validated questionnaire. No incentives (monetary or otherwise) were given to the participants.

### Measurements

All participants completed 1 “Ambulatory Monitoring Wearability Assessment Questionnaire” for each device tested. Data collected included participant demographics and device details (eg, sex, age, device used), the perceived exertion while performing “activities of daily living” (ADLs) using the Borg CR-10 scale [23], the perception of pain or discomfort in specific body areas using body maps with the Borg CR-10 scale [23], and thoughts and feelings about emotions, anxiety, harm, etc, caused by the AMS by using the Comfort Rating Scale (CRS), as described in Multimedia Appendix 2 (CRS information [19] and an open comment section for participants to share general feedback).

The CRS [19] uses a 21-point scale throughout 14 statements, split into 6 categories; 3 statements for emotion, 4 for attachment, 1 for harm, 2 for perceived change, 1 for movement, and 3 for anxiety. All but one of the 14 statements are negatively worded such that, to strongly disagree with a statement (lower score), is a positive outcome [19]. In the case of the 1 positive statement, the answers were further preprocessed (ie, inverted) to make them homogeneous with the other answers, as previously described in another study [17]. For each participant, the median score was first determined for each questionnaire section. For better interpretation, we have also calculated the percentage of responses within each question/category and colored it according to positive or negative outcome (further information in Multimedia Appendix 2). The median score of all the sections was then computed to determine the participant’s overall median CRS score.

To minimize the risk of missing data, the clinical researchers double-checked all the received questionnaires with the participants when collecting them. To minimize wearability bias between devices, we mixed them and documented the order/combination they were used by participants, introduced a washout period of at least one week before testing another device and checked for any clear bias in the free-text feedback section of the questionnaire.

### Data Analysis

#### Sample Size

Owing to the exploratory pragmatic nature of this study, no sample size calculation was performed. We recruited a convenience sample of 20 healthy volunteers to offer a wide range of experiences with wearability of the test devices.

#### Data Preprocessing

For comparisons, we grouped the chest patches (Peerbridge Cor and VitalPatch) and the pulse oximeters (AP-20, Checkme O2+, Nonin, PC-68B, and Wavelet). This grouping allowed us to conduct separate comparisons, as the selected main measurements from the chest patches are heart rate and respiratory rate, while the pulse oximeters include pulse rate and peripheral capillary oxygen saturations (SpO_2_). It is expected that these 2 types of monitors will be part of the same AMS.

#### Statistical Analysis

Due to the limited sample size and data skewness (normality was assessed using the Shapiro-Wilk test), median and IQRs were reported. Nonparametric tests were used to assess differences between the devices’ CRS scores. The Wilcoxon test was used to compare the median CRS scores of the chest patches. Finally, the Kruskal-Wallis test, followed by post hoc Dunn tests [24] (with Bonferroni correction [25]), was used to compare the median CRS scores of the pulse oximeters. All statistical tests were conducted using R v3.6.1 [26] and the tidyverse package [27].

#### Free Text Analysis

Participants were also encouraged to write free text to describe the problems and challenges they encountered with each device. NVivo 12 (NVivo qualitative data analysis software, QSR International Pty Ltd Version 12, 2018) was used to analyze and collate feedback into common categories. For each category, we included the number of participants with at least one negative comment (eg, for disrupted ADLs, the number of participants who took off the device for ADLs or mentioned that these were disruptive when performing daily tasks is reported). This number was reviewed and agreed by 2 researchers from the study team.

## Results

### Participant Characteristics

**Figure 2:**
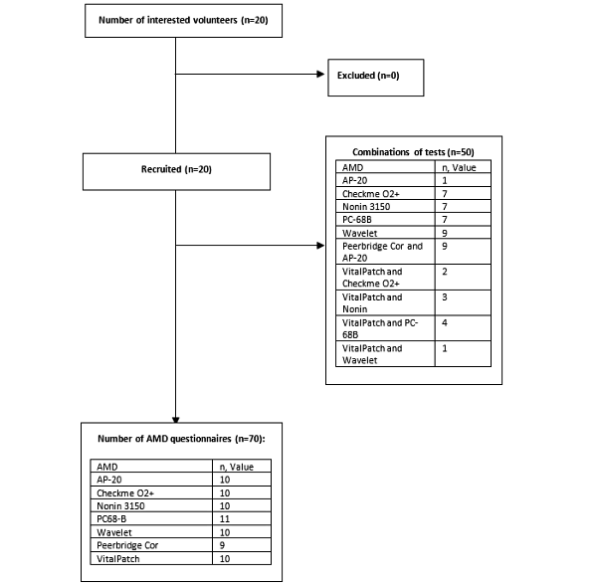
The study participant flowchart. AMD: ambulatory monitoring device.

Twenty in-house volunteers (13 women and 7 men) were recruited between May 4, 2018 and October 30, 2018, with a median (IQR) age of 34 (32-40) years. For each session, participants wore either a pulse oximeter, a chest patch, or both for up to 72 hours before completing the wearability questionnaire. All participants wore at least one device, with a median (IQR) washout period of 31 (13-58) days before wearing another one, and they all completed 1 questionnaire per device (Figure 2).

### Wearability Questionnaire Outcomes

The total wear duration and total number of removals per device are presented in Table 1. Wavelet and Checkme O2+ were the most used pulse oximeters (644/720, 89.4%) and the VitalPatch was the most used chest patch (663/720, 92.1%). These devices also had the lowest pain/discomfort and median exertion scores in the included activities as described in Table 2.

**Table 1:** Device testing duration, removal duration, and reasons for removal.

Measurements		Pulse oximeters										Chest patches				
		AP-20 (n=10)		Checkme O2+ (n=10)		Nonin (n=10)		PC68-B (n=11)		Wavelet (n=10)			Peerbridge Cor (n=9)		VitalPatch (n=10)	
**Testing duration (hours:minutes)**																
	Total planned duration^a^		720:00		720:00		720:00		792:00		720:00			648:00		720:00
	Used duration^b^ (% of total planned duration)		522:31 (72.6)		643:35 (89.4)		590:11 (82.0)		558:43 (70.6)		640:45 (90.0)			491:19 (75.8)		662:52 (92.1)
	Median session duration (IQR)		70:45 (51:50-72:00)		70:40 (58:17-72:00)		64:42 (53:46-69:55)		67:50 (36:58-70:44)		72:00 (55:11-72:00)			70:45 (45:10-71:55)		69:58 (68:12-72:00)
**Removals**																
	Number of participants (total removals)		8 (36)		10 (45)		9 (49)		8 (31)		3 (4)			4 (8)		0
	Median removal duration^c^ (IQR)		1:06 (0:30-5:37)		1:21 (0:50-3:30)		2:01 (1:04-5:05)		01:20 (0:35-3:15)		1:50 (1:33-3:00)			0:10 (0:10-0:45)		0:00 (0:00-0:00)
**Reasons for removal (n)**																
	Hygiene		10		6		6		1		0			6		0
	Discomfort		3		3		7		6		0			0		0
	Cooking or eating		6		6		11		10		0			0		0
	Exercise		0		3		3		4		0			0		0
	Work/social		2		5		4		3		0			0		0
	Battery/hardware failure		10		17		2		2		3			0		0
	Other/unknown		5		5		16		5		1			2		0

^a^Total planned duration refers to the total amount of time if all participants wore the respective device for the full 72 hours (Total=72 × n). Values are shown as hours:minutes.

^b^Used duration: reflects the actual time that the devices were worn by the participants, with missing times representing a combination of device removal periods and differences between the actual end of the session and 72 hours (ie, when the full 72 hours are not achieved). Values are shown as hours:minutes.

^c^Values are shown as hours:minutes.

**Table 2:** Pain/discomfort and exertion scores per device, body part, and activity by using Borg CR-10 scale [23].

Device		Pulse oximeters					Chest patches		
		AP-20	Checkme O2+	Nonin	PC68-B	Wavelet		Peerbridge Cor	VitalPatch
Median pain/discomfort score per body part^a^ (IQR)		3.00 (5.00)	1.50 (2.38)	3.50 (2.50)	3.00 (2.50)	0.75 (0.50)		2.00 (6.00)	1.00 (0.88)
**Median exertion score per activity**									
	Walking	0	0	0	0	0		0	0
	Eating	3	2	3	4	0		0	0
	Drinking	1	0	1	3	0		0	0
	Dressing	5	4	5	8	1		0	0
	Writing	1	0	3	5	0		0	0
	Using phone/tablet	0	0	3	3	0		0	0
	Reading	0	0	0	0	0		0	0
	Hand washing	9	6	7	10	0		0	0
	Sleeping	4	2	3	4	0		3	0

^a^For the pulse oximeters, the body part of interest was the nondominant wrist and for the chest patches, it was the chest.

Common problems identified in free text sections of the questionnaires are presented in Table 3. We grouped free text comments into 5 categories: (1) device size (eg, device being too big or bulky), (2) disrupted ADLs (eg, limited daily tasks, so needed to be removed), (3) skin irritation (eg, some concerns of wrist strap/finger-probe becoming itchy), (4) finger probe uncomfortable (eg, sweaty and annoying), and (5) affected sleep (eg, participants kept waking up and unable to sleep with it on).

**Figure 3:**
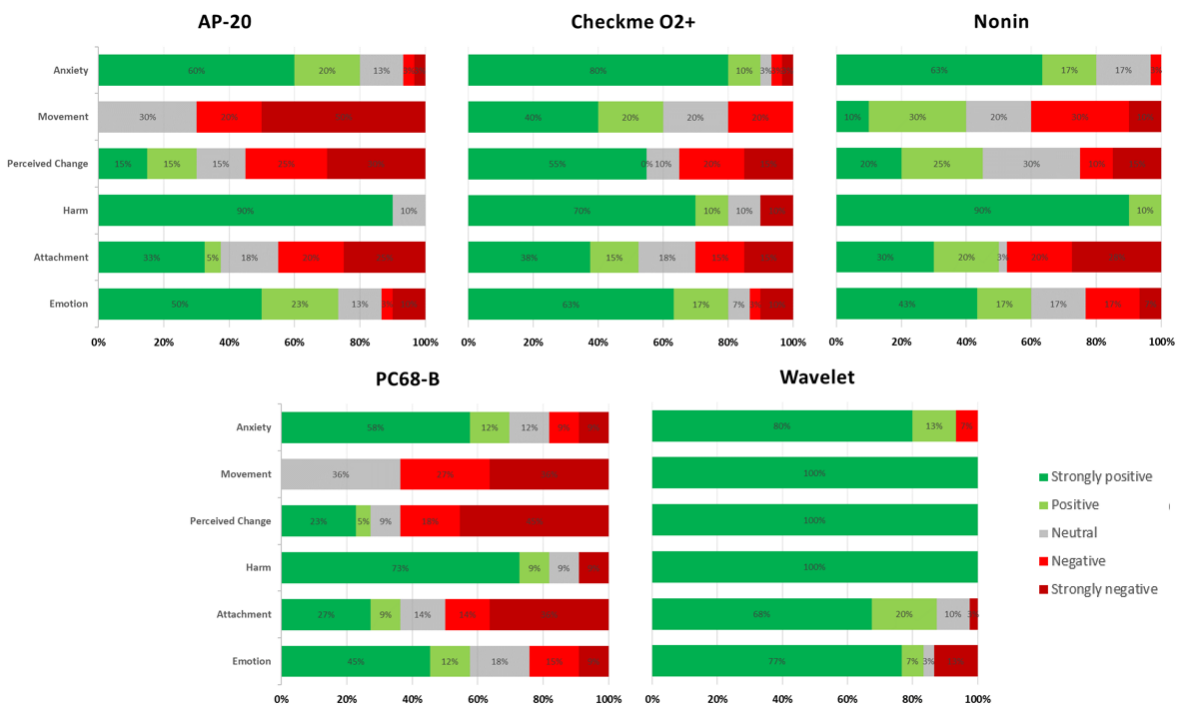
Comfort Rating Scale scores for each pulse oximeter. Green represents the percentage of positive outcomes and red represents the percentage of negative outcomes from the Comfort Rating Scale scores (Multimedia Appendix 2).

**Figure 4:**
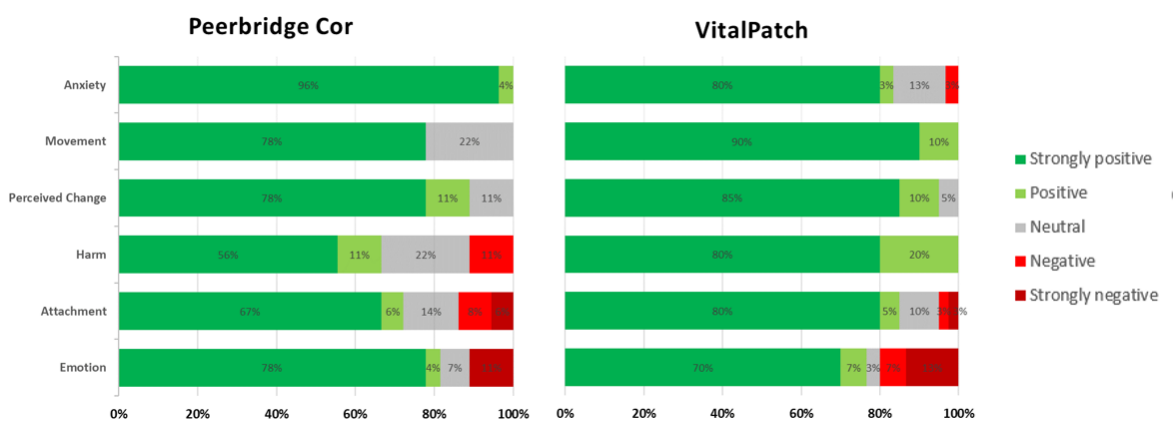
Comfort Rating Scale scores for each chest patch device. Green represents the percentage of positive outcomes and red represents the percentage of negative outcome from the Comfort Rating Scale scores (Multimedia Appendix 2).

Significant differences for most sections were found between the CRS scores of the pulse oximeters as well as between the overall median CRS scores (Table 4 and Table 5). Figure 3 and Figure 4 represent the percentage of positive/negative CRS score outcomes per section and per device.

**Table 3:** Device specifications and participant-identified problems.

Measurements and problems		Pulse oximeters					Chest patches	
		AP-20	Checkme O2+	Nonin	PC-68B	Wavelet	Peerbridge Cor	VitalPatch
Device measurements		SpO_2_^a^, PR^b^, RR^c^, perfusion index	SpO_2_, PR, steps	SpO_2_, PR	SpO_2_, PR, perfusion index	SpO_2_, PR^b^	HR^d^, RR, ECG^e^	HR, RR, ECG, body position
**Participant-identified problems (n)^f^**								
	Device size	2	0	3	10	0	16	0
	Disrupted ADLs^g^	8	6	5	6	0	0	0
	Skin irritation	0	2	1	0	2	3	3
	Finger probe uncomfortable	6	0	5	8	N/A^h^	N/A	N/A
	Affected sleep	1	0	3	5	0	5	1

^a^SpO_2_: oxygen saturation.

^b^PR: pulse rate.

^c^RR: respiratory rate. For the AP-20, respiratory rate is only possible using a nasal cannula.

^d^HR: heart rate.

^e^ECG: electrocardiogram. Peerbridge Cor uses a 2-lead ECG and VitalPatch a single-lead ECG.

^f^The cells show the number of participants identifying at least one problem in each category.

^g^ADLs: activities of daily living.

^h^N/A: not applicable.

**Table 4:** Comparison of the Comfort Rating Scale scores across different pulse oximeters.

CRS^a^ section	Pulse oximeters, median (IQR) score					
	AP-20 (n=10)	Checkme O2+ (n=10)	Nonin (n=10)	PC68-B (n=11)	Wavelet (n=10)	*P* value
Overall score	7.50 (4.00)^b^	3.25 (8.63)^b^	6.00 (6.38)^b^	9.00 (4.75)^b^	1.00 (0.88)^c,d,e,f^	<.001
Emotion	4.00 (6.00)	4.00 (7.25)	6.50 (12.25)^b^	7.00 (5.5)^b^	1.00 (1.00)^e,f^	.02
Attachment	10.75 (5.13)^b^	8.50 (9.75)	10.75 (2.00)^b^	14.00 (3.25)^b^	2.5 (3.00)^c,e,f^	.001
Harm	1.00 (1.75)	1.50 (6.75)	1.00 (0.00)	2.00 (5.00)	1.00 (1.50)	.30
Perceived change	13.75 (4.25)^b^	6.75 (9.13)	10.50 (3.88)	15.00 (6.50)^b^	1.25 (1.38) ^c,f^	<.001
Movement	17.00 (6.00)^b,d^	7.00 (6.75)^g^	10.50 (8.00)^b^	16.00 (7.00)^b^	1.00 (1.00)^c,e,f^	<.001
Anxiety	2.50 (7.75)	1.50 (2.75)	5.00 (6.50)	4.00 (5.50)	1.00 (1.00)	.25

^a^CRS: Comfort Rating Scale.

^b^Statistically significant difference from Wavelet.

^c^Statistically significant difference from AP-20.

^d^Statistically significant difference from Checkme O2+.

^e^Statistically significant difference from Nonin.

^f^Statistically significant difference from PC-68B.

**Table 5:** Comparison of the Comfort Rating Scale scores between the chest patches.

CRS^a^ section	Chest patches, median (IQR)		
	Peerbridge Cor (n=9)	VitalPatch (n=10)	*P* value
Overall score	1.50 (1.50)	1.25 (2.00)	.60
Emotion	1.00 (2.00)	1.00 (1.00)	.92
Attachment	4.50 (2.50)	3.00 (1.00)	.04
Harm	3.00 (10.00)	2.50 (3.50)	.48
Perceived change	2.50 (3.00)	1.00 (3.13)	.17
Movement	3.00 (3.00)	1.00 (1.50)	.06
Anxiety	1.00 (0.00)	1.50 (3.50)	.22

^a^CRS: Comfort Rating Scale.

A post hoc analysis showed that the Wavelet had significantly better scores than other pulse oximeters in most sections of the CRS (*P*<.05), for example, in the CRS total score, the Wavelet was statistically superior to AP-20 (*P*=.004), Checkme O2+ (*P*=.048), Nonin (*P*=.02), and PC-68B (*P*<.001). The Checkme O2+ also showed a significantly better score for Movement against the PC-68B (*P*=.048) and close to statistical significance against the AP-20 (*P*=.05) (Multimedia Appendix 3).

For the chest patches, although there was a statistically significant difference in the Attachment section, no difference was found in the overall median CRS scores between the VitalPatch and Peerbridge Cor (Table 5). In the open feedback, the main difference reported by participants was related to sleep, with the Peerbridge Cor more frequently reported to be uncomfortable.

## Discussion

### Main Results

Twenty participants were recruited for this study, with 70 questionnaires completed (approximately 10 per device) to assess AMS wearability. The Wavelet was found to be the most comfortable wearable pulse oximeter, with the absence of a finger probe, thereby contributing to its better wearability score. Having a probe on the fingertip seemed to negatively impact a device’s wearability, with participants reporting a feeling of tightness and sweatiness after prolonged and continuous use. The finger probe was also often reported as hindering function, requiring its removal to perform activities and affecting the total time the device was worn. Amongst the pulse oximeters with a finger probe, the Checkme O2+ was preferred, probably due to the smaller and ring-shaped finger probe, with placement away from the fingertip. For the remaining pulse oximeters (AP-20, PC-68B, and Nonin), no significant differences were found; however, PC-68B was the most negatively commented on, as participants consistently described this device as bulky and disruptive to ADLs. In addition, it had the most negative feedback for a finger probe, in comparison with AP-20 and Nonin.

For the chest patches, there were only significant differences between the 2 selected devices in favor of the VitalPatch in the Attachment section of the CRS scale. Participants noted that they found it difficult to sleep on their front or side with the Peerbridge Cor.

Preference for the Wavelet, Checkme O2+, and VitalPatch was also reflected in the pain/discomfort score, activity exertion score, and free text feedback given. Participants also reported preference for smaller devices, both for the pulse oximeters and the chest patches. To our knowledge, this is the first study comparing wearability for a number of wearable devices.

### Study Limitations

A clear limitation of this study was the recruitment of in-house healthy volunteers; thus, our results may not reflect the hospitalized population. However, this was the first step within our project to provide evidence on the wearability and to select feasible devices to be further tested. As there was a limited number of devices available, the order was not randomly assigned; these were allocated to participants as they were available, with the order being documented.

Another limitation was that not all participants were able to test all 7 devices, with an average of 3.5 devices used per participant (1 being a chest patch) between all sessions. To avoid bias, participants were encouraged to assess each device individually and not by comparison with previous devices worn. Additionally, although a log of temporary removals was provided, not all participants were fully compliant with it, which may explain the variability in the device removal numbers.

Furthermore, no specific instruction was given to participants regarding the finger probe placement for any of the pulse oximeters (we note that Viatom Technology recommends using the Checkme O2+ on the thumb [28]), and participants were therefore not asked to indicate which finger they used. This could provide an additional comparison between pulse oximeters that use finger probes and such a comparison will be included in future wearability studies.

We have used the CRS scale [18,19] for the main assessment of device wearability in healthy volunteers; however, the 6 domains evaluated and their distribution might not be the most appropriate method to evaluate wearability for clinical monitoring devices, as it does not necessarily ensure a correct representation of and applicability to the clinical environment.

### Comparison With Prior Work

Our questionnaire methodology for wearability evaluation has been described in previous studies [17,18] and adapted here to compare our selected devices using similar outcomes such as the CRS scale, which is designed to assess comfort over a range of dimensions [29]. Wearability has a direct impact on system usability and its clinical implementation, as patients will be more likely to wear the AMS if they feel comfortable, thus improving data availability and quality [30]. A recent review analyzed the validation, feasibility, clinical outcomes, and costs of 13 different wearable devices and concluded that these were predominantly in the validation and feasibility testing phases [31]. Despite the exponential growth in wearable technology, little evidence is available regarding wearability and acceptance in the clinical setting [30,32,33]. We note that, for all the devices under test, only the VitalPatch had indexed wearability studies available [34,35].

This exploratory study is embedded in a comprehensive research project, which aims to test, refine, and deploy these devices in clinical practice. This project follows a human-centered design process that requires a full exploration of the environment into which the technology is to be placed and understanding the eventual end users of the technology [21]. Although this was not tested on end users, our study was the most ethically sound surrogate, as it did not expose patients to equipment that does not work or that they would find intolerable because of discomfort.

The findings of this study will support the initial selection of wearable devices for the next phases of our project for testing the reliability, accuracy, and functionality of the selected devices [22], as it is not known the AMSs that are the most reliable for use in the hospital environment. Once the initial devices have been selected, tested, and refined, patients will also have the opportunity to provide both qualitative and quantitative data on the wearability of the devices.

### Conclusions and Future Research

Our results suggest that traditional pulse oximeter finger probes hinder function, as participants preferred the wrist-worn (Wavelet) and ring-style pulse oximeters (Checkme O2+). The smaller chest patch (VitalPatch) was found to be less noticeable and more comfortable. These preferences were reflected in the total time participants wore the device. These results help to inform which wearable device designs are more likely to be deployed successfully within the hospital environment.

## Appendix

Multimedia Appendix 1 STROBE checklist.

Multimedia Appendix 2 Comfort Rating Scale information.

Multimedia Appendix 3 Post hoc Dunn tests with Bonferroni Correction for Pulse Oximeters.

## Abbreviations

ADLs: activities of daily living
AMS: ambulatory monitoring system
CRS: comfort rating scale
NIHR: National Institute for Health Research
SpO_2_: oxygen saturation
