# Supplementary material for: Wearability Testing of Ambulatory Vital Sign Monitoring Devices: Prospective Observational Cohort Study
Source: JMIR Mhealth Uhealth. 2020 Dec 16;8(12):e20214. doi: 10.2196/20214 (PMC7773507; doi:10.2196/20214)
Supplement: Multimedia Appendix 2 [file mhealth_v8i12e20214_app2.docx]

**Multimedia Appendix 1**: Comfort Rating Scale information.

The comfort rating scale (CRS) was developed as a quick and easy 21-point scale to assess wearability; this tool provides information by measuring comfort in 6 dimensions. Below we outline each dimension and questions of this scale as per previous studies:

**Emotion –** Concerns about appearance and relaxation:

E1.1- I feel worried and embarrassed.

E1.2- I feel tense.

E1.3- I would wear the device if it was invisible.

**Attachment**—Comfort related to non-harmful physical sensation of the device on the body (e.g. the feel of the device either directly as pressing on the body or indirectly as it pulls on clothing or moves in relation to the body):

A2.1- I feel the device on the body.

A2.2- I feel the device moving.

A2.3- I was not able to move as usual.

A2.4- I have difficult in putting on the device.

**Harm**—Physical sensation conveying pain:

H3.1- The attached device causes me some kind of harm.

**Perceived change**—Non-harmful indirect physical sensation making the wearer feel different overall with perceptions such as being awkward or uncoordinated, may result in making conscious compensations to movement or actions:

PC4.1- I feel more bulky.

PC4.2- I feel change in the way people look at me.

**Movement**—Conscious awareness of modification to posture or movement due to direct impedance or inhibition by the device:

M5.1- The device obstructs my movements.

**Anxiety**—Worries as to the safety of wearing the device and concerns as to whether the wearer is using it correctly or it is working appropriately:

AN6.1- I do not feel secure with the device.

AN6.2- I feel that I do not have the device properly attached.

AN6.3- I feel that the device is not working properly.

For Figures 2 and 3 we have calculated the percentage of participants scoring inside each band:

Dark green: 1-5 (strongly disagree)

Green: 6-9 (disagree)

Grey: 10-13 (neither)

Red: 14-17 (agree)

Dark Red: 18-21 (strongly agree)

**References:**

1. Cancela J, Pastorino M, Tzallas AT, Tsipouras MG, Rigas G, Arredondo MT, et al. Wearability assessment of a wearable system for Parkinson's disease remote monitoring based on a body area network of sensors. Sensors (Basel) 2014 Sep 16;14(9):17235-17255 [FREE Full text] [doi: 10.3390/s140917235] [Medline: 25230307]
2. Knight JF, Baber C. A tool to assess the comfort of wearable computers. Hum Factors 2005;47(1):77-91. [doi: 10.1518/0018720053653875] [Medline: 15960088]
