# Supplementary material for: Wearability Testing of Ambulatory Vital Sign Monitoring Devices: Prospective Observational Cohort Study
Source: JMIR Mhealth Uhealth. 2020 Dec 16;8(12):e20214. doi: 10.2196/20214 (PMC7773507; doi:10.2196/20214)
Supplement: Multimedia Appendix 3 [file mhealth_v8i12e20214_app3.docx]

**Multimedia Appendix 2**. Post hoc Dunn tests with Bonferroni Correction for Pulse Oximeters.

Table 6 - Post hoc Dunn test with Bonferroni correction with Bonferroni correction for pulse oximeters, reported P value

| CRS Total Score | | | | | | | |  |
| --- | --- | --- | --- | --- | --- | --- | --- | --- |
|  | | **AP-20** | **Checkme O2+** | **Nonin** | | **PC-68B** | |  |
| Checkme O2+ | | *1.00* | *-* | *-* | | *-* | |  |
| Nonin | | *1.00* | *1.00* | *-* | | *-* | |  |
| PC-68B | | *1.00* | *1.00* | *1.00* | | *-* | |  |
| Wavelet | | *0.004* | *0.048* | *0.02* | | *<0.001* | |  |
| CRS Section: Emotion | | | | | | | | |
| Checkme O2+ | *1.00* | | *-* | | *-* | | *-* | |
| Nonin | *1.00* | | *1.00* | | *-* | | *-* | |
| PC-68B | *1.00* | | *1.00* | | *1.00* | | *-* | |
| Wavelet | *0.44* | | *0.06* | | *0.02* | | *0.01* | |
| CRS Section: Attachment | | | | | | | | |
| Checkme O2+ | *1.00* | | *-* | | *-* | | *-* | |
| Nonin | *1.00* | | *1.00* | | *-* | | *-* | |
| PC-68B | *1.00* | | *0.34* | | *0.98* | | *-* | |
| Wavelet | *0.01* | | *0.13* | | *0.03* | | *<0.001* | |
| CRS Section: Harm | | | | | | | | |
| Checkme O2+ | *1.00* | | *-* | | *-* | | *-* | |
| Nonin | *1.00* | | *1.00* | | *-* | | *-* | |
| PC-68B | *1.00* | | *1.00* | | *0.33* | | *-* | |
| Wavelet | *1.00* | | *1.00* | | *1.00* | | *0.54* | |
| CRS Section: Perceived Change | | | | | | | | |
| Checkme O2+ | *0.60* | | *-* | | *-* | | *-* | |
| Nonin | *0.86* | | *1.00* | | *-* | | *-* | |
| PC-68B | *1.00* | | *0.38* | | *0.57* | | *-* | |
| Wavelet | *<0.001* | | *0.10* | | *0.06* | | *<0.001* | |
| CRS Section: Movement | | | | | | | | |
| Checkme O2+ | *0.054* | | *-* | | *-* | | *-* | |
| Nonin | *0.72* | | *1.00* | | *-* | | *-* | |
| PC-68B | *1.00* | | *0.048* | | *0.70* | | *-* | |
| Wavelet | *<0.001* | | *0.21* | | *0.01* | | *<0.001* | |
| CRS Section: Anxiety | | | | | | | | |
| Checkme O2+ | *1.00* | | *-* | | *-* | | *-* | |
| Nonin | *1.00* | | *1.00* | | *-* | | *-* | |
| PC-68B | *1.00* | | *0.93* | | *1.00* | | *-* | |
| Wavelet | *0.61* | | *1.00* | | *0.44* | | *0.27* | |
